# Emerging functions of lycopene in the management of digestive premalignant lesions

**DOI:** 10.3389/fphar.2024.1478170

**Published:** 2024-10-17

**Authors:** Kerui Gan, Wenjin Shi, Xiangfei Liu, Wei Ding, Yan Qiu, Xiaobo Luo

**Affiliations:** ^1^ State Key Laboratory of Oral Diseases, National Clinical Research Center for Oral Diseases, Chinese Academy of Medical Sciences, Research Unit of Oral Carcinogenesis and Management, West China Hospital of Stomatology, Sichuan University, Chengdu, Sichuan, China; ^2^ Department of Pathology, West China Hospital, Sichuan University, Chengdu, China

**Keywords:** Lycopene, digestive precancerous lesions, oxidative stress, inflammatory response, chemoprevention

## Abstract

Common digestive precancerous lesions, including oral potentially malignant disorders (OPMDs), gastric ulcers and colorectal adenoma, harbor high risk of cancerous transformation. Early intervention of these lesions is significant to prevent carcinogenesis and improve patients’ prognosis. Lycopene, a carotenoid predominantly accumulated in tomatoes, is clinically recommended with its cis structure; as lycopene harbors the most potent antioxidative effects among carotenoids, its chemopreventive effects on the premalignant lesions is noted. Despite several reviews have assessed lycopene’s efficacy for OPMDs, emerging studies have reported varying efficacy for digestive precancerous lesion with no comprehensive summary. Therefore, this review initially evaluates the efficacy and underlying mechanisms of lycopene for management of digestive precancerous lesions. According to the included studies, lycopene may show high promise in the management of digestive precancerous lesions, such as relieving mouth opening and burning sensation of oral submucous fibrosis (OSF), presenting potentially equivalent efficacy on managing oral lichen planus (OLP) as steroids and alleviating gastrointestinal precancers’ symptoms, meanwhile lowering colon cancer risk. Moreover, its mechanisms for managing digestive precancerous lesions are concretely summarized, including anti-oxidative stress effects, anti-inflammatory response and regulation of cell proliferation and apoptosis, especially its modifications on TLR4/TRIF/NF-κB signaling pathway and p53-dependent cell cycle control and apoptosis. More studies are warranted to confirm its long-term efficacy and preventive role against malignant transformation of digestive precancerous lesions as evidence is insufficient.

## 1 Introduction

Digestive cancers pose a significant threat to patients’ life, usually along with poor prognosis. According to Global Cancer Observatory (GCO), colorectal cancer ranked as the fourth most prevalent malignancy worldwide in 2022, while stomach cancer and oral cancer ranks sixth and 17th respectively. Moreover, the estimated mortality rates of 2.4% and 5.7% for cancers of the lip and oral cavity and the esophagus respectively, and 8.4% along with 11.5% individually for stomach and colorectum cancers, indicated that digestive cancers serve as huge burden for human health (Global Cancer Observatory, 2022). Notably, early intervention in the precancerous stage of digestive cancers could significantly reduce the risk of malignant transformation and improve the life quality of patients (Kumari et al., 2022; Kizhakkoottu and Ramani, 2024; Choi et al., 2024). As for oral potentially malignant disorders (OPMDs) which has an overall malignant transformation rate of 7.9%, early prevention and intervention of OPMDs is of great importance to minimize the risk of malignant transformation (Kumari et al., 2022).

Precancerous lesions, also known as premalignant lesions, are epithelial aberrant alterations that possess the potential to progress into cancers if left untreated. Oral precancerous lesions are usually referred to as OPMDs, which refer to a spectrum of oral mucosal disorders that carry an increased risk of malignant transformation into oral squamous cell carcinoma (OSCC), including oral leukoplakia (OLK), oral submucous fibrosis (OSF), oral lichen planus (OLP), etc. (Warnakulasuriya et al., 2021). OPMDs severely impact the quality of life for patients and can even be life-threatening. For example, the main clinical manifestation of OSF is a burning sensation and intolerance to spices, and as the disease progresses, patients experience reduction in mouth opening, which not only causes pain, poor oral hygiene and impaired chewing and swallowing, but also affects patients’ speaking and appearance, causing psychological issues and limitations in social activities (Peng et al., 2020). Similarly, common types of gastric premalignant lesions are comprised of gastric ulcers, gastric polyps, atrophic gastritis, intestinal metaplasia, etc. (Wang et al., 2022). As for colorectum, aberrant crypt foci (ACF) are the earliest precursor to colorectal cancer (CRC), and colorectal adenoma is the most frequent precancerous lesion; besides, other less-frequent colorectal premalignant lesions cover inflammatory bowel disease (IBD) and some hereditary syndromes (Alrawi et al., 2006; Conteduca et al., 2013; Orlando et al., 2008).

Lycopene is one sort of non-provitamin A and liposoluble carotenoid, acting as a vital part of the human diet. As it could not been synthesized by human body, the primary intake of which is from dietary sources such as ripe tomatoes, pink guavas and several other red fruits and vegetables (Bramley, 2000), with at least 85% of the lycopene in the human diet deriving from tomatoes and tomato products (Bramley, 2000). The red carotenoid is a straight-chain unsaturated alkene with the chemical formula C40H56 that can exist in both cis and trans isomers. While predominantly serving as all-trans isomer in natural plants, the majority of lycopene in human plasma and tissues is cis-isomer, which has superior polarity and water solubility than all-trans isomer (Gärtner et al., 1997; Cooperstone et al., 2015). As a result, compared to the all-trans isomer, the cis form may be better absorbed through the digestive tracts. *In vitro*, the configuration transformation from trans-isomer to cis-isomer can be accomplished through light, heat and specific chemical processes; *in vivo*, gastrointestinal and liver enzymes are the main agents responsible for isomerization (Ross et al., 2011). Owing to its liposoluble and isomeric characters, lycopene is more bioavailable in processed tomato products such as ketchup than in raw tomatoes, especially when the carotenoid is processed with oil (Fielding et al., 2005; Tan et al., 2010). Therefore, cis-isomerized lycopene is recommended for clinical use. Lycopene has been utilized as a plant pigment due to its characteristic of red color, however, its potent antioxidant activity and chemopreventive effect have gained increasing attention in recent years.

So far, accumulating evidences have indicated the positive effects of lycopene in preventing cervical and prostate cancers (Mirahmadi et al., 2020). For instance, an umbrella meta-analysis published in 2024 revealed that dietary lycopene is reversely associated with the risk of prostate cancer, digestive cancer and head and neck cancer (Sui et al., 2024). The therapeutic effects of lycopene for head and neck and digestive cancers have also been recognized in the literatures (Al-Ishaq et al., 2020; Zanoni et al., 2019). Besides, as mentioned above, early detection or intervention is crucial in minimizing the possibility of the malignant tumor formation (Kumari et al., 2022; Kizhakkoottu and Ramani, 2024; Choi et al., 2024). Hence, it is important to explore various modalities and mechanisms of preventing and treating digestive precancerous lesions. Notably, emerging researches have suggested the efficacy and mechanisms of lycopene in managing precancerous lesions of the digestive tract including OPMDs, ulcerative colitis, etc. (Kumar et al., 2007; Patil et al., 2014; Singh et al., 2014; Subramaniam et al., 2014; Goel and Ahmed, 2015; Nayak, 2015; Patil et al., 2015; Kopuri et al., 2016; Saran et al., 2018; Piyush et al., 2019; Johny et al., 2019; Arakeri et al., 2020; Gupta et al., 2021; Singh et al., 2004; Singh and Bagewadi, 2017; Kushwaha, 2017; Eita et al., 2021; Hazzaa et al., 2021; Jain and Katti, 2015; Głąbska et al., 2016; Głąbska et al., 2019; Tekeli et al., 2019; Yin et al., 2023; Jung et al., 2013), with rare side effects, which may correlate with its antioxidative mechanisms (Eita et al., 2021; Tekeli et al., 2019; Yin et al., 2023; Boyacioglu et al., 2016; Jain et al., 1999; Aljawad et al., 2014; Li et al., 2021; Sengupta et al., 2006; Liu et al., 2006; Vrieling et al., 2007); one study of meta-analysis in 2020 incorporating 7 RCTs of OSF reported that lycopene is only effective in refining maximum mouth opening without improving other symptoms (Guo et al., 2020); while another study including relevant studies up to April of 2022 indicated that lycopene was capable of relieving symptoms of mouth opening and tongue protrusion of patients with OSF, which meanwhile reducing pain and promoting resolution of OLP, exhibiting comparable efficacy as steroids (Al-Maweri et al., 2023); therefore, conflicting efficacy of lycopene for managing OPMDs was implied. Meanwhile, as digestive cancers pose heavy health burden globally, no relevant review assessing the efficacy of lycopene for digestive precancers has been proposed. Therefore, it is warranted for us to update the previous reviews of OPMDs and gastrointestinal premalignant lesions, and comprehensively assess and explore the potential role of lycopene in managing these digestive precancerous lesions.

## 2 Efficacy of lycopene in digestive precancerous lesions

In recent years, numerous studies have evaluated the efficacy of lycopene in managing precancerous lesions of the digestive tract, and relevant clinical or animal studies over the last 10 years have been summarized and shown in Table 1.

**TABLE 1:** General characteristics of included literatures evaluating efficacy of lycopene on digestive precancerous lesions.

Study	Study design	Type of precancerous lesions	Subject	Intervention groups			Control groups			Outcomes assessment	Follow-up	Side effects of lycopene	Main findings
				Number of patients (M/F)	Age of patients	Formulation	Number of patients (M/F)	Age of patients	Formulation				
Kumar, 2007, India	RCT	OPMD (OSF)	Human	Group A: n = 21 Group B: n = 19	Group A: median age = 28 Group B: median age = 26	Group A: lycopene 16 mg daily in 2 equal doses for 2 months Group B: lycopene 16 mg daily in 2 equal doses + betamethasone intralesional injections biweekly for 2 months	Group C: n = 18	Group C: median age = 29	Group C: placebo for 2 months	Mouth opening; Visual inspection; Palpatory findings; Burning sensation	6 months	No lycopene associated side effects	Significant improvements of mouth opening were observed in Group A and B comparing with Group C. The improvement was better in Group B than in Group A but the difference is not significant
Patil, 2014, India	RCT	OPMD (OSF)	Human	n = 34	Mean age = 30.9	lycopene 4 mg twice a day for 3 months	n = 34	Mean age = 30.9	Spirulina 500 mg in 2 divided doses for 3 months	Mouth opening; Lesions (ulcers/vesicles/erosions); VAS	2 months	No obvious side effects	Significant improvement of mouth opening was seen in lycopene group, while spirulina set showed significant refinement of ulcers/erosions/vesicles
Singh,2014, India	RCT	OPMD (OSF)	Human	n = 22	Mean age = 29.41	10 mg lycopene capsules daily in two equally divided doses for 2 months	n = 22	Mean age = 25.59	Betamethasone 4 mg/mL intralesional injection twice a week for 2 months	Mouth opening; VAS	NA		A significant increase in mouth opening and relief of burning sensation was observed in both groups, and the improvement was better in lycopene set.
Subramaniam, 2014, India	RCT	OPMD (OSF)	Human	n = 15	Not mention	16 mg lycopene capsules daily in two equally divided doses for 6 weeks	n = 15	Not mention	5 min ultrasound to both cheeks each for 15 days	Mouth opening; Tongue protrusion; VAS	3 months	Not reported	Lycopene significantly improved burning sensation and tongue protrusion
Goel, 2015, India	RCT	OPMD (OSF)	Human	Lycopene group: n = 90 (223/47); Betamethasone group n = 90 (223/47)	Mean age = 31	Lycopene group 2 mg lycopene capsules twice a day for 6 months Betamethasone group: betamethasone 4 mg/mL intralesional injection biweekly for 6 months	n = 90 (223/47)	Mean age = 31	No treatment	Mouth opening	NA	Not reported	Lycopene significantly increased mouth opening. Notably, lycopene was more effective than betamethasone in stage III.
Nayak,2015, India	RCT	OPMD (OSF)	Human	Lycopene group: n = 24; Lycopene + Vit. E group: n = 24	Mot mention	Lycopene group 8 mg lycopene capsules daily in two equal doses for 3 months; Lycopene + Vit. E group:8 mg lycopene capsules daily in two equal doses +400 I.U. vitamin E + 0.2 mg selenium in two equal doses for 3 months	n = 24	Not mention	Placebo once a day 3 months	Mouth opening; VAS; Lesions (erythematous areas/ulceration/erosions)	2 months	Not reported	Lycopene in combination of vitamin E was significantly more effective than lycopene alone in OSF management
Patil, 2015, India	RCT	OPMD (OSF)	Human	n = 60	Mean age = 31.6	8 mg lycopene capsules daily in two equally divided doses for 3 months	n = 60	Mean age = 31.6	Aloe vera 5 mg topically applied thrice a day for 3 months	Mouth opening; Tongue protrusion; VAS	NA	Not reported	Improvements of mouth opening and tongue protrusion were significant in lycopene group
Kopuri, 2016, India	RCT	OPMD (OSF)	Human	n = 15	>15 years	8 mg lycopene capsules daily in two equally divided doses for 3 months	n = 15	>15 years	800 mg curcuma daily in two equally divided doses for 3 months	Mouth opening; Burning sensation; Pain; Submucosal layer thickness	NA	Not reported	Lycopene was more effective than curcumin in the treatment of OSF.
Saran, 2018, India	RCT	OPMD (OSF)	Human	n = 30	Mean age = 26.00	4 mg lycopene capsules daily in two equally divided doses for 3 months	n = 30	Mean age = 27.90	300 mg curcumin thrice daily for 3 months	Mouth opening; VAS	NA	Not reported	Lycopene was more effective than curcumin in improvement of mouth opening
Piyush, 2019, India	RCT	OPMD (OSF)	Human	Curcumin group: n = 30 Lycopene group: n = 30	Age range = 17–60	Curcumin group: curcumin and piperine 300 mg, 1 tablet twice a day for 6 months; Lycopene group: 8 mg lycopene capsules, 1 tablet twice a day for 6 months	n = 30	Age range = 17–60	Placebo capsule once a day for 6 months	Mouth opening; VAS; Tongue prostrusion; Cheek flexibility	NA	No obvious side effects	There was significant improvement in mouth opening, tongue protrusion, cheek flexibility and burning sensation for both curcumin and lycopene groups
Johny,2019, India	RCT	OPMD (OSF)	Human	Group A: n = 15 Group B: n = 15	Not mention	Group A: 16 mg lycopene capsules daily in two equally divided doses for 6 months Group B: 16 mg lycopene capsules daily in two equally divided doses +1500 IU hyaluronidase intralesional injection twice a week for 6 months	n = 15	Not mention	Placebo 6 months	Mouth opening VAS Visual and palpatory findings	NA	Not reported	A significant improvement of burning sensation and mouth opening was detected in both lycopene and lycopene–hyaluronidase group compared with placebo group
Arakeri, 2020, India	RCT	OPMD (OSF)	Human	Group A/B: n = 200 (200/0) (After 1 year follow-up, Group A were equally divided into A1 and A2)	Mean age = 29.9	Group A: 8 mg lycopene capsules daily in two equally divided doses for 3 months; Group A1: retreatment at 1 year follow-up; Group A2: Observation at 1-year follow-up	n = 200 (200/0)	Mean age = 28.8	Placebo 3 months	Mouth opening VAS	3 years in total	No obvious side effects	A significant improvement of burning sensation and mouth opening was observed in Group A. The long-term preventive effect of lycopene was seen in Group A1
Gupta,2021, India	RCT	OPMD (OSF)	Human	n = 15	Not mention	20 mg Tretiome per day for 1 month	n = 15	Not mention	20 mg lycopene capsules daily in two equally divided doses for 1 month	VAS; Speech and swallowing problems; Xerostomia Movement of fibrotic areas; Development of pain and soreness	1 month	Not reported	There was significant improvement of mouth opening and decrease in burning sensation in Tretiome group compared with lycopene group
Singh,2004, India	RCT	OPMD (OLK)	Human	Group A: n = 20 (15/5) Group B: n = 20 (14/6)	Group A: 10-70 years (65% in 31-60 years); Group B: 10-70 years (80% in 31-60 years)	Group A: 8 mg lycopene daily in two equally divided doses Group B: 4 mg lycopene daily in two equally divided doses 3 months	Group C: n = 18	Group C: range at 10–70 (72.22% in 31-60 years)	Placebo 3 months	Clinical response: bi-dimensional measurement of the lesions and color photography Histological response: histological examination and grading of dysplasia	2 months	Not reported	Lycopene significantly improved both the clinical and histological changes compared to placebo. The efficacy increased with elevating dose
Singh,2017, India	RCT	OPMD (OLK)	Human	n = 30 (28/2)	Range at 26–75 (50.00% in 56-65 years)	*Calendula officinalis* gel (containing 2 mg/g antioxidants) for 3 months	n = 30 (22/8)	Range at 26–75 (53.33% in 56-65 years)	Lycopene gel (containing 2 mg/g lycopene) for 3 months	Clinical response: size of the lesions	NA	Not reported	Both lycopene and *Calendula officinalis* were significantly effective in reducing the size of lesion
Kushwaha, 2017, Nepal	RCT	OPMD (OLP)	Human	n = 13 (7/6)	Mean age = 41	2 mg lycopene capsules twice a day for 8 weeks	n = 15 (3/12)	Mean age = 48	Prednisolone 40 mg/d for 8 consecutive weeks, then 30 mg/d for 2 weeks, 20 mg/d for next 2 weeks and finally 10 mg/d for 2 weeks	NRS; Severity of lesion (REU)	NA	Flatulence and nausea	Significant improvements of burning sensation and severity of lesion were observed in both lycopene and corticosteroid group, but corticosteroid was more potent than lycopene
Eita, 2021, Egypt	RCT	OPMD (OLP)	Human	n = 10 (4/6)	Mean age = 51.5	lycopene 10 mg/d 8 weeks	n = 10 (2/8)	Mean age = 45.9	Prednisolone 40 mg/d for 4 weeks, decreasing by 10 mg/d per week for the next 3 weeks and by 5 mg/d for the last week	NRS; Lesion activity (Escudier scores); Serum concentrations of 8-isoprostane	NA	Not reported	A significant improvement was observed in both groups. Lycopene significantly reduced the serum levels of 8-isoprostane
Hazzaa, 2021, Egypt	RCT	OPMD (OLP)	Human	n = 20 (8/12)	Mean age = 52.1	lycopene 10 mg twice a day for 8 weeks	n = 20 (8/12)	Mean age = 52.1	Prednisolone 40 mg/d for 8 weeks, then 30 mg/d for 2 weeks, 20 mg/d for next 2 weeks and finally 10 mg/d for 2 weeks	VAS Clinically (score); Salivary MDA level	NA	NA	Lycopene was significantly effective in the reduction of pain, mucositis and salivary MDA level, similar to corticosteroid
Jain, 2015, India	RCT	GU	Rats	Group II: n = 6 Group III: n = 6 Group IV: n = 6	NA	Group II: 100 mg/kg hesperidin Group III: 2 mg/kg lycopene Group IV: 2 mg/kg lycopene and 100 mg/kg hesperidin	Group I: n = 6 Group V: n = 6	NA	Group I: 10 mL/kg vehicle (2% gum acacia solution) Group V: 10 mg/kg omeprazole	Gastric pH, total acidity, ulcer index, macroscopic examination	NA	NA	Both lycopene and hesperidin significantly reduced the volume of gastric content and total acidity as well as increase gastric pH. The combination of two drugs was more effective
Glabska, 2016, Poland	Cross-sectional study	UC	Human	NA	NA	NA	NA	NA	NA	The correlation between fecal blood, fecal mucus, abdominal pain, fecal pus and intake of lycopene	NA	NA	Higher lycopene intake in patients with ulcerative colitis in remission was associated with lower fecal blood occurrence
Glabska, 2019, Poland	Cross-sectional study	UC	Human	NA	NA	NA	NA	NA	NA	The correlation between the incidence of bowel movements, flatulence, constipation, painful tenesmus and intake of total lycopene	NA	NA	Intake of lycopene was not correlated with any of the gastrointestinal symptoms included in the study
Tekeli, 2019, Turkey	RCT	UC	Rats	Group S: n = 7 Group LC: n = 7 Group TCL: n = 7	NA	Group LC: 10 mg/kg lycopene daily Group TLC: 10 mg/kg colon targeted lycopene daily 7 days treatment +4% acetic acid at day 7	Group C: n = 7	NA	Group C: only 4% acetic acid at day 7 Group S: 100 mg/kg sulfasalazine daily for 7 days +4% acetic acid at day 7	Histopathological examination	NA	Not reported	Pre-administration with colon targeted lycopene was significantly potent
Yin, 2023, China	RCT	UC	Rats	Group II: n = 10 Group III: n = 10 Group IV: n = 10 Group V: n = 10	NA	Group II: 1 mL/kg olive oil once a day (vehicles) with 0.5 mg/kg OTA Group III: vehicles with 0.5 mg/kg OTA and 100 mg/kg quercetin Group IV: vehicles with 0.5 mg/kg OTA and 100 mg/kg lycopene Group V: vehicles with 0.5 mg/kg ochratoxin A, 100 mg/kg quercetin and 100 mg/kg lycopene 7 days	Group I: n = 10	NA	Group I: vehicles 7 days	Colitis: ulcerogenic characteristics, DAI score Histological study Gene expression	NA	Not reported	Lycopene significantly reduced DAI score and colonic damage of rats with OTA-induced UC.
Jung, 2013, USA	Cohort study	Colorectal adenomas	Human	NA	NA	NA	NA	NA	NA	Dietary intake of carotenoids of participants Type, location, size and histology of polyps	1986–2006	NA	Lycopene intake was inversely related to the risk of colorectal adenomas

Abbreviations: RCT, randomized controlled trial; GU, gastric ulcers; UC, ulcerative colitis; NA., not available; DAI, disease activity index; MDA, malondialdehyde; NO, nitric oxide; SOD, superoxide dismutase; GSH, glutathione; MMP-7, matrix metalloproteinase 7; SFFQ, semi-quantitative food frequency questionnaire; OPMD, oral potentially malignant disorders; OLK, oral leukoplakia; OLP, oral lichen planus; NRS, numeric rating scale; REU, reticular, erythematous, and ulceration; VAS, visual analogue scale; OSF, oral submucous fibrosis; USG, ultrasonography; OTA, ochratoxin A.

### 2.1 Lycopene for the management of oral potentially malignant disorders

OSF is one of the most common OPMDs closely related to the consumption of betel nuts (Warnakulasuriya and Chen, 2022). After reviewing these relevant studies, 13 studies demonstrated that lycopene could significantly refine the clinical manifestations of OSF patients, among which 12 randomized controlled trial (RCT) studies have compared the potential efficacy between lycopene and other treatment modalities or the combinational effectiveness of lycopene with other drugs, apart from one study by Arakeri et al. which focused on the long-term efficacy of lycopene rather than its comparison with other therapeutic reagents (Kumar et al., 2007; Patil et al., 2014; Singh et al., 2014; Subramaniam et al., 2014; Goel and Ahmed, 2015; Nayak, 2015; Patil et al., 2015; Kopuri et al., 2016; Saran et al., 2018; Piyush et al., 2019; Johny et al., 2019; Arakeri et al., 2020; Gupta et al., 2021).

Glucocorticoids, either topical or oral administration, has long been utilized for treating OSF due to its anti-inflammatory effects. Two studies have explored the various efficacy between lycopene and betamethasone for the treatment of OSF(23,25). Singh’s study including 44 patients demonstrated that lycopene was superior than intralesional injection of betamethasone in improving mouth opening and burning sensation (Singh et al., 2014). However, another study dividing 270 OSF patients into 3 different clinical stages and the effectiveness was assessed in various stages; specifically, lycopene capsules and intralesional injected betamethasone were comparatively effective in increasing the mouth opening in patients at stage I, while betamethasone was more effective than lycopene in stage II, by contrast, lycopene was more potent than betamethasone in stage III. The distinct results may be correlated with varied accessibility of betamethasone for patients at different clinical stages with various degree of mouth opening (Goel and Ahmed, 2015). Apart from the above comparative studies, the RCT study of Kumar and colleagues investigated the efficacy about the combination of lycopene and betamethasone contrasted to the single application of lycopene. The results suggest that although the improvement of mouth opening was better in combination group, the difference was insignificant (Kumar et al., 2007). More RCTs with larger population exploring the combination of lycopene with steroids are required for further evidence of the combined application, thus lowering the side effects of steroids with less needed dose.

Besides, three studies have analyzed and compared the therapeutic effects between lycopene and curcumin (Kopuri et al., 2016; Saran et al., 2018; Piyush et al., 2019). Kopuri’s study revealed that lycopene possessed better performance in improving mouth opening and fibrous bands whereas curcumin mainly contributed to better refining in burning sensation and blanching, concluding that lycopene was more effective than curcumin on the treatment of OSF(28). Similarly, another study indicated that lycopene was more efficacious in increasing mouth opening than curcumin, while none significant relief of burning sensation was indicated between these two drugs (Saran et al., 2018). Besides, Piyush and colleagues have reported the equivalent therapeutic efficacy between lycopene and curcumin for OSF(30).

Additionally, in comparison to aloe vera which is another antioxidant, lycopene exhibited better therapeutic effects in treating OSF (Patil et al., 2015; Sudarshan et al., 2012). Patil et al. discovered that lycopene exhibited a greater effect regarding the enhancement of mouth opening, while spirulina was more advantageous in reducing ulcers (Patil et al., 2014). In a single-blinded RCT, the researchers revealed that Tretiome was superior to lycopene as for the refinement of mouth opening and burning sensation (Gupta et al., 2021). Moreover, several studies have examined the combinatorial efficacy of lycopene with other drugs for OSF. One study suggested that the combination of lycopene and vitamin E may remarkably improve the clinical symptoms of OSF patients than lycopene alone (Nayak, 2015). However, when lycopene was used in combination with hyaluronidase, the effect was comparative as that of lycopene alone (Johny et al., 2019).

While the above studies assessed the efficacy of lycopene in comparison to other drugs or their combination for OSF, the study by Subramaniam et al. investigated the various effectiveness between lycopene and a non-pharmacological approach, therapeutic ultrasound, for managing OSF, suggesting that lycopene has better performance in improving burning sensation and tongue protrusion (Subramaniam et al., 2014). Notably, Arakeri et al. have evaluated the long-term efficacy of lycopene in OSF. Specifically, part of the participants received a second intervention 1 year after the initial treatment of 3 consecutive months. After 1 year of follow-up post re-treatment, recurrence of symptoms was observed among the participants without re-treatment, whereas the clinical manifestations of retreated patients were as that in the initial treatment. Besides, no lycopene related-side effects were observed during the whole trial. Therefore, re-treatment with lycopene at 1 year after the onset of its initial therapy was beneficial in preventing the recurrence of the symptoms (Arakeri et al., 2020).

Hence, based on findings from several RCTs, Lycopene is efficacious in improving mouth opening, tongue protrusion, and burning sensation of OSF compared with placebo; besides, the combination of lycopene with vitamin E might achieve better outcome, so the combined application of lycopene with other strategies are warranted to be confirmed in more well-designed RCTs. Moreover, re-treatment with lycopene is recommended to prevent the symptoms from recurring.

OLK is one common type of OPMDs whose overall malignant transformation rate is estimated to be 3.5% (Aguirre-Urizar et al., 2021). Singh et al. examined the therapeutic effects of lycopene on OLK, concluding that it could significantly refine both clinical signs and histological progression of OLK lesions compared to placebo; and positive correlation was observed between the efficacy and the dosage of drug (Singh et al., 2004). Another study comparing the efficacy between lycopene gel and Calendula officinalis gel for OLK showed that lycopene had similar effectiveness as the other gel, both of which significantly reduced the size of OLK (35). In all, more well-designed RCT is required to validate the efficacy of lycopene for preventing malignant transformation and reducing lesion size of OLK.

OLP is typically treated with topical steroids, with systemic corticosteroids being a common recommendation for resistant patients; however, the application of steroids carry multiple risks, such as adrenal suppression, Cushing’s syndrome and immunosuppression (Liu et al., 2013; K et al., 2011). Three studies examined the therapeutic effects of lycopene on OLP and compared the efficacy between lycopene and prednisolone (Kushwaha, 2017; Eita et al., 2021; Hazzaa et al., 2021). One study showed that, although lycopene significantly reduced clinical symptoms such as burning sensation and severity of lesion in patients with OLP, prednisolone was more effective than lycopene; besides, the lycopene group experienced side effects such as flatulence and nausea, while the prednisolone group reported side effects such as facial puffiness and dizziness (Kushwaha, 2017). However, in two other RCTs, the lycopene group exhibited a statistically significant decrease in pain and lesion severity, and lycopene showed comparable efficacy to that of prednisolone (Eita et al., 2021; Hazzaa et al., 2021). As no lycopene-related side effects were reported in either study, Eita et al. reported occurrence of facial puffiness, gastrointestinal disorders and weakness associated with prednisolone (Eita et al., 2021; Hazzaa et al., 2021). Thus, lycopene serves as a potent alternative when steroids is unsuitable.

As the study on OLP or OLK are still too limited to support the therapeutic role of lycopene, more well-designed studies with long-term observation are required to further uncover the potential.

### 2.2 Lycopene for the management of gastrointestinal precancerous lesions

Up till now, a few studies have confirmed the potential of lycopene in the treatment of certain gastrointestinal precancerous lesions (Jain and Katti, 2015; Głąbska et al., 2016; Głąbska et al., 2019; Tekeli et al., 2019; Yin et al., 2023; Jung et al., 2013). Jain et al. have performed one RCT exploring the effectiveness of lycopene and hesperidin on pyloric ligation-induced gastric ulcer in rats (Jain and Katti, 2015). The results implied that, although the combination of lycopene and hesperidin exhibited better efficacy, lycopene alone could already markedly reduce the volume of gastric contents, total acidity, and elevate the gastric pH (39).

Ulcerative colitis (UC), one of the dominant forms of inflammatory bowel disease, is associated with an increased risk of developing CRC (9). A cross-sectional study by Glabska et al. analyzed the correlation between the diet of UC patients in remission and their gastrointestinal symptoms, indicating that a higher consumption of lycopene was correlated with a lower incidence of fecal blood in these patients, whereas another similar study by the same research group reported no significant association between lycopene intake and the decreased occurrence of constipation in UC patients (Głąbska et al., 2016; Głąbska et al., 2019). The results of these two trials suggest that increasing the intake of lycopene may alleviate certain gastrointestinal symptoms and improve the overall wellbeing of UC patients, although further prospective studies with large samples are required to confirm this conclusion. The protective effect of lycopene against UC has also been confirmed in animal studies. One, conducted by Yin et al., revealed that lycopene significantly decreased the colonic damage caused by ochratoxin A (OTA)-induced UC(43). Another study that examined the comparative protective effects of conventional and colon-targeted lycopene on acetic acid-induced UC revealed that UC colonic damage, such as ulceration and hemorrhage, was significantly improved in the colon-targeted lycopene group compared with the acetic acid-induced UC group, while no significant changes were observed in conventional lycopene, although there were signs of improvement (Tekeli et al., 2019).

Colorectal adenomas are precancerous lesions of nearly all sporadic CRC (9). In a cohort study with a total of 29,363 patients, out of whom 3,997 were diagnosed with colorectal adenoma, the association between risk of colorectal adenomas and intake of specific carotenoids was analyzed, showing that lycopene intake was negatively correlated with adenoma risk (Jung et al., 2013).

Thus, unlike these studies performed in OPMDs, the studies investing the efficacy of lycopene on managing gastrointestinal precancerous lesions were predominantly limited to animal studies, observational studies, or cohort studies, RCTs evaluating on its efficacy are needed in the future.

## 3 Possible mechanisms of lycopene in managing premalignant lesions of digestive tract

### 3.1 Antioxidative mechanisms

**FIGURE 1:**
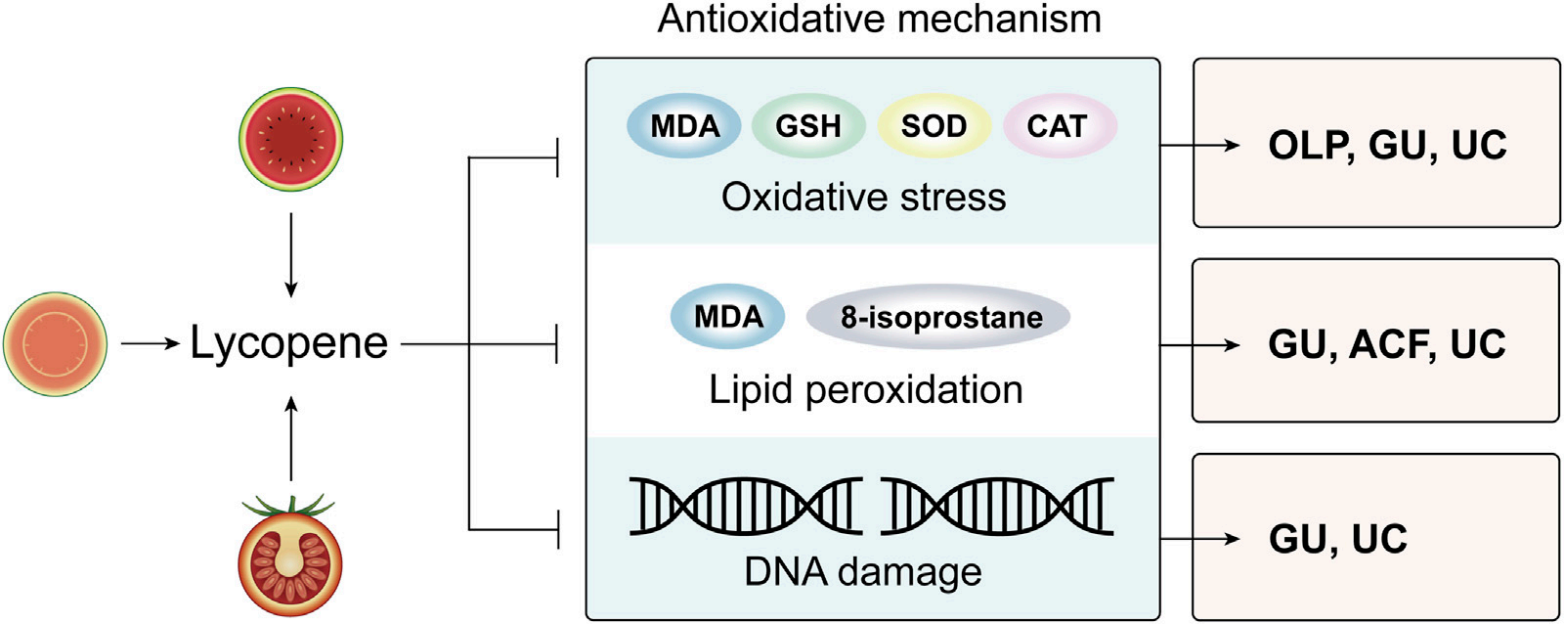
An overview of antioxidative mechanism of lycopene in managing premalignant lesions of digestive tract. Abbreviations: MDA: malondialdehyde; GSH: glutathione; SOD: superoxide dismutase; CAT: catalase; GU: gastric ulcers; ACF: aberrant crypt foci; UC: ulcerative colitis; OLP: oral lichen planus.

Lycopene is a potent antioxidant and has strong capacity to protect against oxidative damage, therefore playing a crucial role in treating selected disorders associated with oxidative stress, including some of the precancerous lesions (Figure 1).

As immune system is reported to play primary role in the pathogenesis and carcinogenesis of OLP, free radicals and ROS may intensify the dysfunction of the immune system (Fu et al., 2000). 8-isoprostane serves as a reliable biomarker of oxidative stress (Amirchaghmaghi et al., 2016). The result of one RCT showed significant decrease of serous 8-isoprostane following 8 weeks of lycopene administration, suggesting that lycopene’s effects on OLP might be associated with its ability to reduce oxidative stress (Eita et al., 2021).

Gastric ulcers can be triggered by a variety of factors, including nonsteroidal anti-inflammatory drugs (NSAIDs) and alcohol. Boyacioglu et al. conducted an RCT to investigate the prophylactic effects of lycopene against indomethacin-induced gastric ulcer in rats (Boyacioglu et al., 2016).The results showed that pre-administration of lycopene could protect against DNA damage induced by indomethacin, possibly by improving the superoxide dismutase (SOD) activity, glutathione (GSH) level and decreasing catalse (CAT) activity, malondialdehyde (MDA) level and myeloperoxidase (MPO) activity, thus exerting its antioxidative effects. Another study also confirmed the anti-lipid peroxidation capacity of lycopene on azoxymethymethane (AOM) induced- ACF which is one of the preneoplastic lesions of colorectal carcinoma (Jain et al., 1999).Aljawad et al. investigated the preventive effect of both Vanadyl sulfate and lycopene in comparison to Lansoprazole in ethanol–induced gastric ulcer and concluded that these two drugs exert a positive effect through antioxidative stress (Aljawad et al., 2014).

Studies have indicated a strong correlation between oxidative stress and the progression of UC (Zhou et al., 2018). Li et al. investigated the prophylactic effect and mechanism of lycopene in dextran sulfate sodium (DSS)-induced UC, and they revealed that compared to DSS group, the clinical symptoms of mice in lycopene pre-administrated group were much better controlled and the level of the antioxidant enzymes and lipid peroxidation were significantly decreased (Li et al., 2021). Another study found that lycopene significantly reduced levels of MDA, nitric oxide (NO), MPO and hydroxyproline and increase levels of SOD and GSH, thereby inhibiting oxidative stress and lipid peroxidation and exerting therapeutic effect in ochratoxin A (OTA)-induced UC rats (Yin et al., 2023). Moreover, regarding that GSH is critical in DNA synthesis, while DNA damage is correlated with UC, we can conclude that lycopene exerts its beneficial effect on UC by preventing oxidative-related DNA damage (Yin et al., 2023; Zhang et al., 2024).

### 3.2 Anti-inflammatory response

#### 3.2.1 TLR4/TRIF/NF-κB signaling pathway

It is reported that the progression of UC is closely associated with the TLR4/NF-κB signaling pathway which has emerged as a vital target for therapeutic interventions aimed at preventing of UC development. Once toll-like receptor 4 (TLR-4) is activated, Tir domain-containing adaptor inducing interferon-beta (TRIF) can bind to TRAF6, then activate a series of kinases, promote entry of activated nuclear factor-kappa B (NF-κB) into nucleus and activate the transcription factor, ultimately leading to the expression and release of inflammatory cytokines. Li et al. found that lycopene pre-administration could modulate the TLR4/TRIF/NF-κB pathway by significantly downregulating the expressions of TLR-4, TRIF, phosphorylated p65 proteins, which in turn suppresses the production of inflammatory cytokines IFN-γ, TNF-α, IL-6, and IL-1β in colonic tissue, thereby reducing inflammation response in the context of UC(48). Additionally, the expression of tight junction-associated proteins in colonic tissues was significantly upregulated after the intervention, suggesting that the intervention may attenuate damage to intercellular tight junctions triggered by inflammatory factors, thereby protecting the intestinal mucosal barrier integrity.

#### 3.2.2 Downregulate expressions of cyclooxygenase-2 and inducible nitric oxide synthase

Overexpression of cyclooxygenase-2 (COX-2) and inducible nitric oxide synthase (iNOS) are correlated to colorectal carcinogenesis, and inhibition of their activities leads to cancer prevention (Sheehan et al., 1999; Rao et al., 1999; de Oliveira et al., 2017). Sengupta et al. discovered that lycopene significantly decreased the incidence of AOM-induced ACF, the earliest precursor of CRC, by inhibiting of COX-2 and iNOS, which were overexpressed in the process of colorectal carcinogenesis, thereby preventing the development of CRC (49).

#### 3.2.3 Downregulate expression of NF-κB and upregulate expression of Nrf-2

**FIGURE 2:**
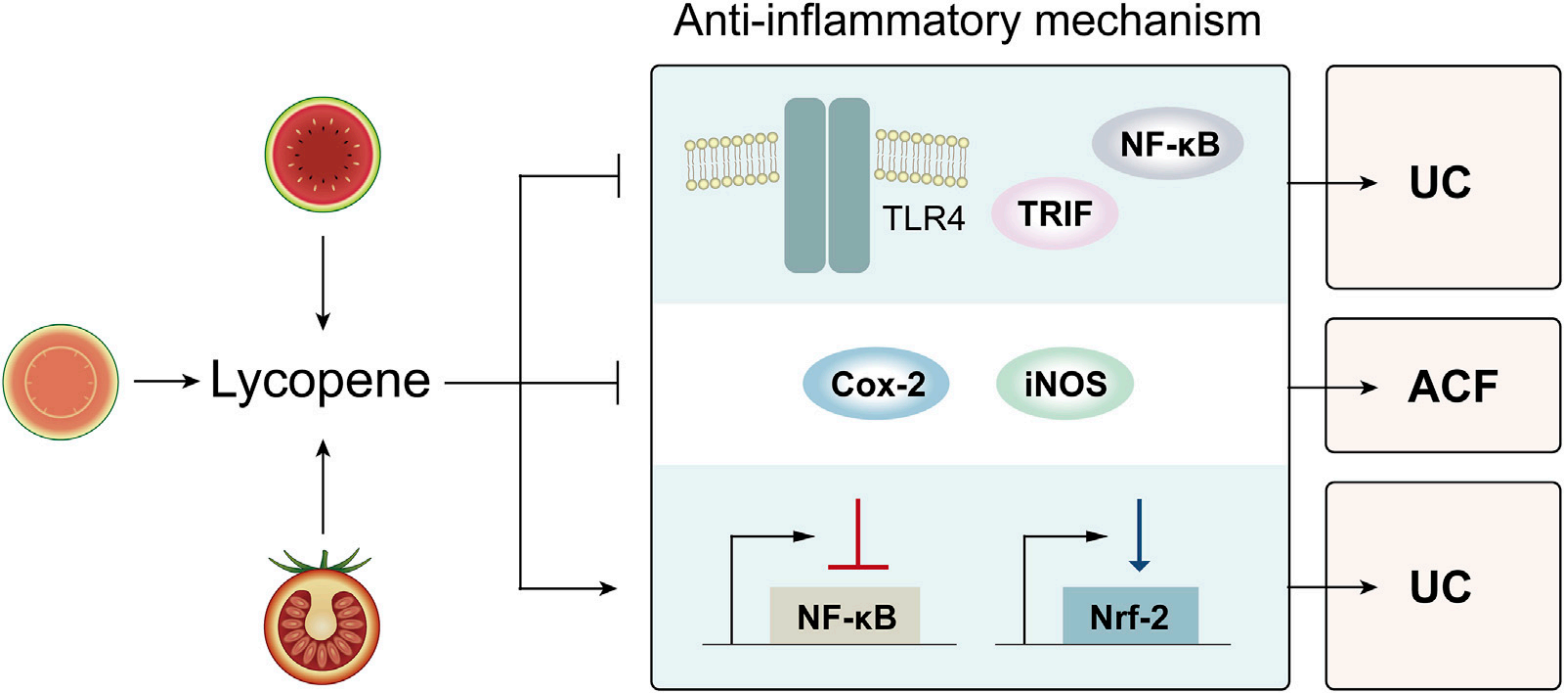
Various anti-inflammatory effects of lycopene in controlling digestive premalignant lesions. Abbreviations: TLR4: toll-like receptor 4; TRIF: Tir domain-containing adaptor inducing interferon-beta; NF-κB: nuclear factor-kappa B; Cox-2: cyclooxygenase-2; iNOS: inducible nitric oxide synthase; Nrf-2: nuclear factor-erythroid 2–related factor 2; UC: ulcerative colitis; ACF: aberrant crypt foci.

NF-κB regulated inflammatory responses and its activation lead to increased expression of pro-inflammatory cytokines, while nuclear factor-erythroid 2–related factor 2 (Nrf-2) reduced the synthesis of inflammatory mediators by inhibiting the NF-κB (Hegazy and El-Bedewy, 2010; Thimmulappa et al., 2016). The study conducted by Tekeli et al. revealed that lycopene significantly decreased levels of NF-κB, IL-1β and IL-6 while increasing the level of Nrf-2, implying that lycopene acts as an anti-inflammatory agent in UC by suppressing the expression of NF-κB and promoting the expression of Nrf-2 (42) (Figure 2).

### 3.3 Anti-cell proliferation and pro-apoptosis effects

#### 3.3.1 p53-dependent cell cycle control and apoptosis

**FIGURE 3:**
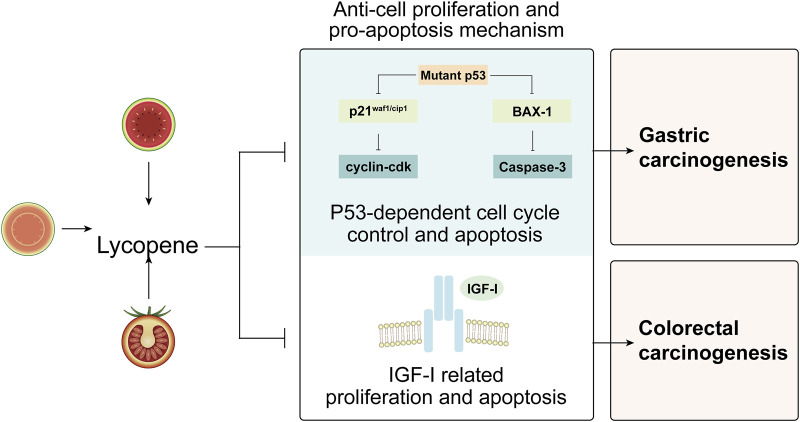
Potential anti-proliferation and pro-apoptosis machinery of lycopene in the management of precancerous lesions of digestive tract. Abbreviations: BAX: BCL2-associated X protein; IGF-I: insulin-like growth factor I.

p53 is a crucial gene that regulates the balance between cell proliferation and apoptosis through a variety of routes by activating its target genes p21^Waf1/Cip1^ and BCL2-associated X protein (Bax)-1. Studies have shown that malfunction and mutation of p53 gene may lead to an escalation in cell proliferation and a reduction in apoptosis, ultimately carcinogenesis, including gastric carcinogenesis (Moss, 1998; Xie et al., 2004). Specifically, under the stimulation of DNA damage, p53 is activated and its target genes p21^Waf1/Cip1^ and Bax-1 are inhibited, and suppression of p21^Waf1/Cip1^ towards cyclin-CDK and proliferating cellular nuclear antigen (PCNA) is diminished, thereby activating the cell cycle. Similarly, when Bax-1 is inhibited, caspase-3 is inactivated. As a result, the balance between cell proliferation and apoptosis is disrupted, contributing to gastric carcinogenesis (El-Deiry, 1998; Isobe et al., 2004; Aoyagi et al., 2003). Study by Liu et al. revealed that lycopene could re-establish this disturbed balance. They showed that smoking increased the expressions of total p53 and phosphorylated p53 in the gastric mucosa, while lycopene supplementation could significantly reversed the aberrant changes; furthermore, lycopene not only prevented the smoking-induced decrease in p21^waf1/cip1^, proapoptotic protein Bax-1 and cleaved caspase 3 but also reversed the increase in the cell proliferation markers including cyclin D1 and PCNA, suggesting that lycopene protects the gastric mucosa from smoking-induced damage through p53-dependent cell cycle control and apoptosis, and may counteract the development of gastric cancer associated with smoking (Liu et al., 2006) (Figure 3).

#### 3.3.2 Insulin-like growth factor I-related proliferation and apoptosis

Higher serum level of insulin-like growth factor I (IGF-I) has been associated with an increased risk of cancer by stimulating proliferation and suppressing apoptosis (Giovannucci et al., 2000; Liu et al., 2003). In a crossover study by Vrieling et al., lycopene was observed to increase the concentrations of circulating insulin-like growth factor binding proteins (IGFBP)-1 and −2, thereby reducing the binding of IGF-I to its receptor, which may indirectly result in decreased bioavailability of IGF-I, potentially playing a preventive role in the development of colorectal cancer (Vrieling et al., 2007) (Figure 3).

## 4 Discussion

In this review, we briefly introduced the natural sources, biosafety and bioactivity of lycopene and summarized its potential efficacy as well as mechanisms underlying its preventive effects on digestive premalignant lesions.

Lycopene is recognized as a safe and potent antioxidant, playing a significant role in the management of precancerous lesions through several mechanisms, including anti-oxidative stress, anti-inflammatory response and regulation of cell proliferation and apoptosis. Lycopene is vital in treating certain digestive precancerous lesions associated with oxidative stress. It exerts anti-inflammatory effects by modulating TLR4/NF-κB signalling pathway and regulation of molecules such as COX-2, iNOS, NF-κB and Nrf-2. Additionally, lycopene corrects the imbalance between cell proliferation and apoptosis by influencing p53 and IGF-I related proliferation and apoptosis.

**FIGURE 4:**
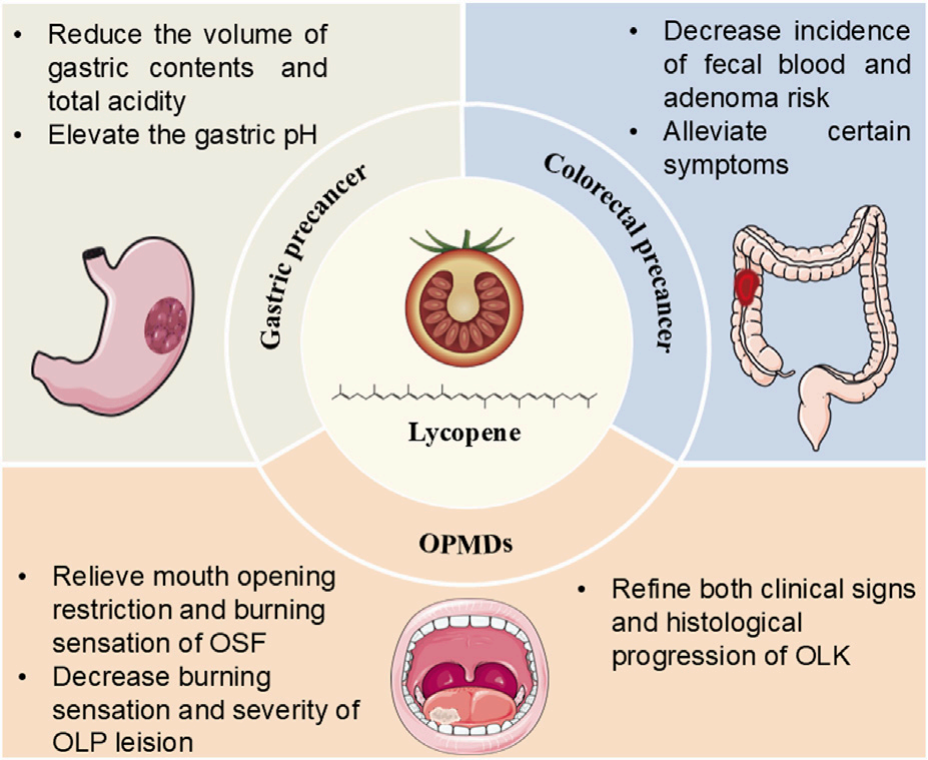
Summary regarding the efficacy of lycopene against digestive premalignant lesions. Abbreviations: OPMDs: oral potentially malignant disorders; OSF: oral submucous fibrosis; OLP: oral lichen planus; OLK: oral leukoplakia.

Most of the RCT studies regarding OSF indicate that lycopene could relieving mouth opening and burning sensation of patients, thereby improving the life quality. However, given the small sample sizes of these RCTs, the generalizability of the findings is limited. In addition, demographic diversity is not mentioned in most of the studies, which may affect the applicability of the findings to various populations. Besides, lycopene might be regarded as a potent alternative strategy for managing OLP when steroids is not appropriate, offering comparative and safer therapeutic effect with less side effects. Although the underlying mechanisms are unclear, Haque et al. revealed that IFN-γ, an anti-fibrotic cytokine, could significantly reverse clinical signs of OSF patients; while in another study, lycopene was observed to suppress hepatic fibrogenesis, suggesting that lycopene may exert its positive effect on OSF by inhibiting fibrosis (Haque et al., 2001; Heber and Lu, 2002). Moreover, lycopene might exhibit preventive potential for managing other digestive precancers including gastric ulcer, UC and colorectal adenomas, suggesting its broad application in digestive premalignant lesions (Figure 4).

For the current research, we offered an exhaustive synthesis of the evidence regarding lycopene’s role in managing digestive precancerous lesions and recommendations are made for the clinical use of lycopene. However, insufficient research is currently available to support the role of lycopene in preventing malignant transformation of digestive lesions, thus, larger sample sizes of human clinical trials are warranted to further uncover the potential role of lycopene in preventing the malignant transformation of digestive lesions. we suggest that the long-term effects of lycopene and its combination with other therapies for digestive premalignant lesions are worth investigating. And studies on the bioavailability of lycopene in different dosage forms may contribute to improve the red carotenoid’s efficacy.

In all, based on our research, it is promising to exploit lycopene as a convenient and efficacious approach for alleviating symptoms and preventing malignant transformation of digestive precancerous lesions as well as improving patients’ life quality and prognosis. We call for more research on the role of lycopene in preventing the malignant transformation of digestive precancerous lesions.
